# Evaluation of Black Soldier Fly (*Hermetia illucens*) Larvae and Pre-Pupae Raised on Household Organic Waste, as Potential Ingredients for Poultry Feed

**DOI:** 10.3390/ani9030098

**Published:** 2019-03-19

**Authors:** Kiyonori Kawasaki, Yuka Hashimoto, Akihiro Hori, Toshiya Kawasaki, Hirofumi Hirayasu, Shun-ichiro Iwase, Atsushi Hashizume, Atsushi Ido, Chiemi Miura, Takeshi Miura, Satoshi Nakamura, Tomohiro Seyama, Yoshiki Matsumoto, Koji Kasai, Yasuhiro Fujitani

**Affiliations:** 1 Graduate School of Agriculture, Kagawa University, Ikenobe 2393, Miki-cho, Kita-gun, Kagawa 761-0795, Japan; kkawasaki@ag.kagawa-u.ac.jp (K.K.); s17g607@stu.kagawa-u.ac.jp (Y.H.); s17g609@stu.kagawa-u.ac.jp (A.H.); s18g619@stu.kagawa-u.ac.jp (T.K.); 2 Research Institute of Environment, Agriculture and Fisheries, Osaka Prefecture, Shakudo 442, Habikino, Osaka 583-0862, Japan; HirayasuH@mbox.kannousuiken-osaka.or.jp (H.H.); IwaseS@mbox.kannousuiken-osaka.or.jp (S.-i.I.); seyama@mbox.kannousuiken-osaka.or.jp (T.S.); kasai@mbox.kannousuiken-osaka.or.jp (K.K.); FujitaniY@mbox.kannousuiken-osaka.or.jp (Y.F.); 3 Graduate School of Agriculture, Ehime University, Tarumi 3-5-7, Matsuyama, Ehime 790-8566, Japan; hashizume.atsushi.ly@ehime-u.ac.jp (A.H.); ido@agr.ehime-u.ac.jp (A.I.); chiemi8@agr.ehime-u.ac.jp (C.M.); miutake@agr.ehime-u.ac.jp (T.M.); 4 Faculty of Environmental Studies, Hiroshima Institute of Technology, Miyake 2-1-1, Saeki-ku, Hiroshima 731-5193, Japan; 5 Japan International Research Center for Agricultural Sciences, Ohwashi 1-1, Tsukuba, Ibaraki 305-8686, Japan; s.nakamura@affrc.go.jp

**Keywords:** black soldier fly, egg quality, microbiota, laying hens, poultry feed, sustainable feed production

## Abstract

**Simple Summary:**

Black soldier fly (BSF) larvae and pre-pupae were raised on nutritionally resembling household organic waste. Next, whole (non-defatted) BSF larvae and pre-pupae were dried and added to the diets of laying hens as a replacement of soybean meal and oil contents. Eggshell thickness and microbiota diversity in the cecum of hens supplemented with BSF pre-pupae showed higher values than those of hens fed with the control diet. It is suggested that chitin, an indigestible substance found in BSF, as well as BSF fat, possibly increased eggshell thickness and microbiota diversity values. Further investigation of the effect of BSF fat added to poultry feed is recommended.

**Abstract:**

Black soldier fly (BSF) larvae and pre-pupae could be satisfactorily raised on household organic waste and used as poultry feed, offering a potential sustainable way to recycle untapped resources of waste. The present study was conducted to determine if whole (non-defatted) BSF larvae and pre-pupae raised on experimental household waste could substitute soybean meal and oil as ingredients for laying hen diets. While no significant differences in feed intake and the egg-laying rate of hens were observed throughout the experiment, egg weight and eggshell thickness were greater in the pre-pupae-fed group than in the other groups. Moreover, although diversity of the cecal microbiota was significantly higher in the pre-pupae-fed than in the control group, no significant differences in bacterial genera known to cause food poisoning were observed when comparing the treatment groups. Nonetheless, *Lactobacillus* and *Bifidobacterium* populations were significantly lower in the treatment than in the control group. Fat content in BSF was possibly related with the changes in the cecal microbiota. Hence, since BSF fat was deficient in essential fatty acids, special attention should be paid to the fat content and its fatty acid composition in the case of regular inclusion of BSF larvae and pre-pupae oil as an ingredient in poultry diets.

## 1. Introduction

Insects, such as black soldier flies (BSF) and their larvae, have been suggested as alternative sources of protein to corn and soybean meals and hence, as potential ingredients for chicken feed [1,2,3,4]. Moreover, authorization of insects as animal protein is expected to be issued for the European poultry industry in 2020–2022 [4].

There are several companies worldwide that produce BSF larvae as feed [5]. Since BSF larvae do not absorb pesticides or mycotoxins [6], BSF larvae are nowadays commercially raised on cereal by-products [4]. Nonetheless, apart from agricultural by-products, larvae could also be raised on animal manure and household organic waste. Although growth performance and meat quality of poultry fed on BSF larvae raised on horse manure have been previously investigated [7], no studies have been conducted to date to test if BSF larvae could be raised on household organic waste and then used as livestock feed due to several reasons, including restriction by sanitary laws and a lack of public acceptance [6]. Nonetheless, using BSF raised on animal manure and household organic waste as animal feed could potentially offer a sustainable way to recycle untapped resources in waste [8].

The use of defatted BSF larvae meal as feed for laying hens has been previously reported [2,3,4,9]. Indeed, feeding defatted BSF meal to laying hens was shown to either increase [10,11] or decrease [12,13] eggshell thickness and eggshell strength. Moreover, BSF larvae meal has been found to contain chitin [14,15], an indigestible substance. When indigestible material reaches the cecum of laying hens it modulates gut microbiota and short chain fatty acids (SCFA) production [15]. Indeed, complete replacement of soybean meal with BSF larvae meal was shown to alter the cecal microbiota in laying hens [15]. Moreover, it was reported that the chitin content of BSF pre-pupae was higher than that of BSF larvae [16], which would likely cause a greater modulation. 

The aim of the present study was to evaluate BSF larvae and pre-pupae raised on experimental household waste as potential ingredients for laying hen diets. To assess this theory, we fed whole BSF larvae and pre-pupae to laying hens, monitored egg quality, and analyzed intestinal tissues, cecal SCFA and the cecal microbiota in both BSF larvae and pre-pupae-fed and control hens.

## 2. Materials and Methods

### 2.1. Production of Black Soldier Flies

Experimental household organic waste (Table 1) was used as BSF larvae feed. BSF eggs were hatched following the methods described by Nakamura et al. [17]. At seven days old, larvae were seeded (1 larva/g) in experimental household organic waste and raised on this material for 10–15 days at 25–30 °C. To remove their tract contents prior to using them as the experimental ingredients for laying hen diets, the obtained larvae and pre-pupae were fasted for 2 days at 25 ± 2 °C.

BSF larvae and pre-pupae were dried at 60 °C for 48 h using a drying oven (DKN 602, Yamato Scientific Co., Ltd., Tokyo, Japan). To analyze for methionine and cystine contents, dried BSF larvae and pre-pupae were oxidized with performic acid. After undergoing hydrochloric hydrolysis, the dried material was then analyzed with an automated amino acid analyzer (L-8900, Hitachi High-Tech Science Corporation, Tokyo, Japan) for proteinogenic amino acid (except tryptophan) content. Tryptophan was measured using high performance liquid chromatography as described by Çevikkalp et al. [18]. Fatty acids were analyzed by gas chromatography according to the AOAC (Association of Official Analytical Chemists) method [19].

### 2.2. Diets

Basal and experimental diets were formulated to compare the effects of replacing soybean meal and oil with BSF larvae and pre-pupae raised on experimental household organic waste in diets of laying hens. The basal diet consisted of maize grain, soybean meal and soybean oil, and was formulated and fed as the control diet. Dried BSF larvae and pre-pupae were pulverized to a size that prevented fat loss and that could be easily mixed with the other feed ingredients. Feed ingredients and the chemical composition of the experimental diets are shown in Table 2. Daily nutrient requirements in the diets were met as according to the recommendations of the National Agriculture and Food Research Organization [20]. Chemical composition of the diets excluding chitin were analyzed according to the AOAC method [19]. Chitin concentration of the diets was calculated as previously described in the literature [4,21].

### 2.3. Animals and Experimental Design

A total of 54 laying hens (Julia; 168 days old) were equally allocated based on their egg-laying rate into P (pre-pupae), L (larvae) and C (control) groups (*n* = 18). Diets and water were offered ad libitum and the duration of the experiment was five weeks. Egg production and egg weight were recorded daily; the egg-laying rate was also calculated on a daily basis.

On the last day of the experiment, hens were sacrificed by decapitation and their duodenum, jejunum, ileum, liver and cecal contents were collected. Moreover, small intestinal tissues and the cecal content of 30 hens (10 per group) whose egg production rate was close to the average value were used for further analysis. Experiments were conducted with the approval of the Kagawa University Animal Experiment Committee (Permission number: 2018-18648).

### 2.4. Villus Height and Crypt Depth

Intestinal samples (0.5 cm) from the duodenum, jejunum, and ileum were fixed by immersion in 4% phosphate-buffered paraformaldehyde for 48 h. These samples were then washed in a phosphate-buffered saline solution and dehydrated by serial immersions in ethanol (70% for 12 h, 80% for 1 h, 90% for 1 h, and 99.5% for 1 h). Finally, intestinal samples were rinsed with xylene, wiped and embedded in paraffin.

Height of villi and depth of crypts in the duodenum, jejunum and ileum were measured using a photomicroscope (Olympus, BX51, Tokyo, Japan) and image analysis software (WinROOF ver 7.4.5, MITANI Corporation, Tokyo, Japan).

### 2.5. Egg Quality

Eggs laid on day 30 of the experiment were used for analysis. The eggs were stored at 20 °C for 24 h after collection and total egg weight, egg yolk weight, albumin weight, egg shell weight, eggshell thickness, eggshell strength, yolk color, yolk height and haugh unit were measured. Eggshell thickness was measured using an eggshell thickness gauge (Eggshell thickness gauge, Fujihira Industry Co., LTD., Tokyo, Japan). Eggshell strength was measured using an automatic egg strength meter (Egg Shell Strength Meter, Fujihira Industry Co., LTD., Tokyo, Japan). Egg weight, yolk weight, albumin weight, eggshell weight, yolk color, yolk height, and haugh unit were measured using an automatic egg quality measuring instrument (EMT-5000, JA ZEN-NOH EGG Co., LTD., Tokyo, Japan).

### 2.6. Calcium, Inorganic Phosphorus and Magnesium Concentration in the Plasma

To obtain the plasma, blood samples were collected at the time of decapitation of hens and were then centrifuged at 500× *g* for 15 min at 4 °C, and stored at −80 °C until further analysis. The concentrations of calcium, phosphorus and magnesium in plasma were measured using a hematology analyzer (Spotchem EZ SP-4430, ARKRAY. Inc., Kyoto, Japan).

### 2.7. SCFA in the Cecal Content

The concentration of SCFA in the cecal contents was measured using high-performance liquid chromatograph (Column: Shim-pack SCR-102H, Detector: Shimadzu CDD10A; Shimadzu Corporation, Kyoto, Japan), as previously described in the literature [22].

### 2.8. PCR Amplicon Sequencing of 16S rRNA Genes

DNA was extracted from the cecal content using a Fast DNA SPIN kit for soil (MP Biomedicals, California, CA, USA), as according to the manufacturer’s instruction. The variable region V3-4 of bacterial 16S rRNA genes was amplified using universal primers 341F [5′-CCTACGGGNGGCWGCAG-3′] and 805R [5′-GACTACHVGGGTATCTAATCC-3′] [23]. The PCR reaction mixture was as follows: 10 µM forward primer, 10 µM reverse primer, 2.5 mM dNTPs, 10× EX buffer, and Ex-taq (TakaRa Bio, Shiga, Japan), pure water and the extracted cecal DNA template. The first PCR conditions were as follows: initial denaturation at 94 °C for 120 s, followed by 25 cycles at 94 °C for 30 s, 55 °C for 30 s and 72 °C for 30 s and a final extension at 72 °C for 5 min. The second PCR conditions were as follows: initial denaturation at 94 °C for 120 s, followed by 12 cycles at 94 °C for 30 s, 60 °C for 30 s and 72 °C for 30 s and a final extension at 72 °C for 5 min. The amplicon was purified using AMPure XP beads (Beckman Coulter, Brea, CA, USA). The quality of libraries was evaluated using a Fragment Analyzer and a dsDNA 915 Reagent Kit (Advanced Analytical Technologies, Inc., Ankeny, IA, USA). Paired-end sequencing for all libraries was conducted with an Illumina MiSeq sequencer (Illumina, San Diego, CA, USA) using a MiSeq Reagent kit v2 (Illumina, San Diego, CA, USA), as per the manufacturer’s instructions.

### 2.9. Taxonomic Analysis Based on 16S rDNA

Chimeric checks were carried out using USEARCH v 8.0 and UCHIME v 4.2.40 software [24]. In addition, creation of operation taxonomic units (OTU) and phylogenetic estimation were conducted using QIIME v1.9 [25]. The database was 97% OTU of Green genes attached to pipeline Qiime for microbiome analysis and all sequences not judged as chimera were extracted and used for subsequent analysis.

### 2.10. Statistical Analysis

Egg laying rate, villus length, crypt depth, egg quality parameters, plasma minerals and cecal SCFA content were compared among the experimental groups by performing the Tukey–Kramer test (SPSS statistic, IBM, New York, NY, USA). Alpha (α) diversity (Chao1 index: richness, Shannon index: evenness) were calculated with a phyloseq package [26] and statistically analyzed using the Tukey–Kramer test. Beta (β) diversity and the UniFrac distance between samples were calculated and statistically analyzed using multivariate analysis (PERMANOVA) and visualized using principal coordinate analysis plots. Plots were generated using Qiime. The relative abundance of fecal microbiota genera was statistically analyzed using the Welch’s *t*-test in STAMP software [27]. Values were considered to be statistically significant when *p* < 0.05. Data are shown as means ± standard errors.

## 3. Results

### 3.1. BSF Larvae and Pre-Pupae Composition

The amino acid and fatty acid profiles of BSF larvae and pre-pupae are shown in Table 3 and Table 4, respectively. Total percentages of amino acids were lower and those of fatty acids higher in BSF larvae than in BSF pre-pupae (Table 3 and Table 4). In addition, total percentages of saturated fatty acids were lower in BSF larvae than in BSF pre-pupae. Nonetheless, BSF larvae had higher percentages of polyunsaturated fatty acids than did BSF pre-pupae, as an effect of the increase levels of linoleic acid (C18:2n-6), an essential fatty acid for poultry (Table 4).

### 3.2. Body Weight, Liver Weight, and Egg-Laying Rate

The temperature of the poultry house was maintained at 24.8 ± 3.4 °C throughout the experiments. There were no significant differences in feed intake, body weight (BW), liver weight, and the egg-laying rate between hen groups (Table 5). In addition, no incidences of mortality were observed during the experiment.

### 3.3. Villus Height, Crypt Depth and Goblet Cell Density

There were no significant differences in the villus height of duodenum, jejunum and ileum, and crypt depth of duodenum, jejunum and ileum between hen groups (Table 6).

### 3.4. Egg Quality

Egg quality parameters are shown in Table 7. While egg weight and albumin weight were significantly higher in the P group than in the other groups (*p* < 0.05), no significant differences in egg yolk weight, egg shell weight, egg shell strength and haugh unit were found among hen groups. Likewise, no significant differences in egg shell thickness were detected between treatment and control groups; however, egg shell thickness was significantly higher in the P group than in the L group (*p* < 0.05). Furthermore, egg yolk color and albumin height were significantly higher in the treatment groups than in the control group (*p* < 0.05).

### 3.5. Concentrations of Calcium, Inorganic Phosphorus and Magnesium in Plasma

The concentration of plasma calcium was significantly higher in the P group than in the L group (Table 8). However, no significant differences were observed in the concentrations of plasma inorganic phosphorus and magnesium among hen groups.

### 3.6. SCFA in the Cecal Content

No significant differences in the cecal acetic acid, propionic acid, n-butyric acid and total SCFA were found among hen groups (Table 9).

### 3.7. Cecal Microbiota

**Figure 1:**
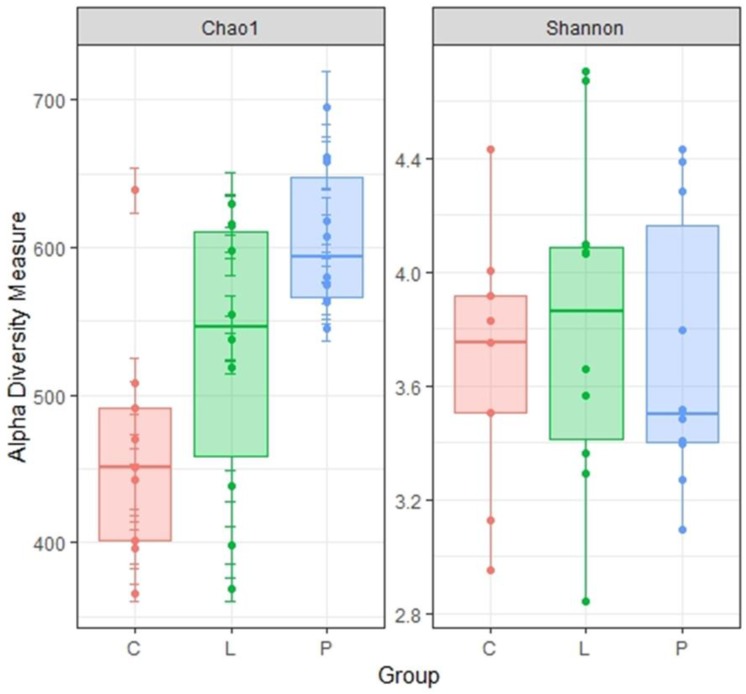
Microbial communities in the gut of experimental hens collected after 5 weeks of BSF larvae and pre-pupae dietary supplementation (C: *n* = 9; L: *n* = 10; P: *n* = 10). α-diversity indices for control and experiment hens. C: control diet; L: larvae diet; P: pre-pupae diet.

A total of 904 OTUs were detected in 1,426,724 sequences, although one library from the control group could not be amplified. When α diversity (Chao 1 index: richness, Shannon index: evenness) in all hen groups was compared, the Chao1 index was found to be significantly higher in the treatment groups than in the control group (*p* < 0.05), but the Shannon index was similar among groups (Figure 1). 

**Figure 2:**
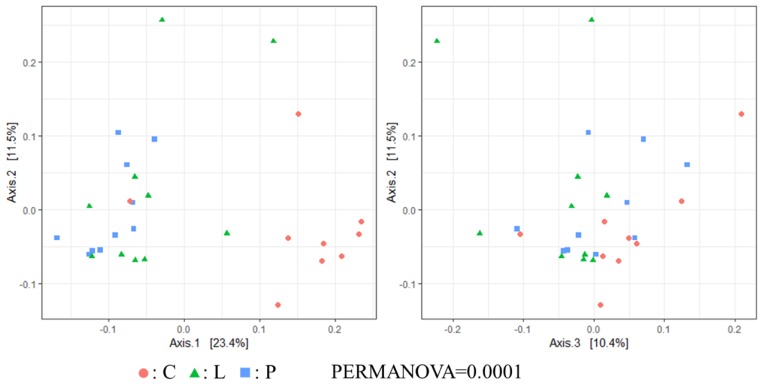
Microbial communities in the gut of experimental hens collected after 5 weeks of black soldier fly (BSF) larvae and pre-pupae dietary supplementation (C: *n* = 9; L: *n* = 10; P: *n* = 10). Unweighted UniFrac distance principal coordinate analysis (PCoA) plots of β-diversity measurement of the microbiota communities. C: control diet; L: larvae diet; P: pre-pupae diet.

**Figure 3:**
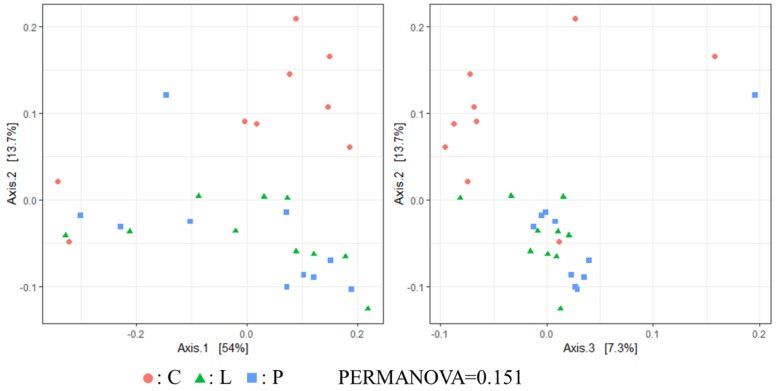
Microbial communities in the gut of experimental hens collected after 5 weeks of BSF larvae and pre-pupae dietary supplementation (C: *n* = 9; L: *n* = 10; P: *n* = 10). Weighted UniFrac distance principal coordinate analysis (PCoA) plots of β-diversity measurement of the microbiota communities. C: control diet; L: larvae diet; P: pre-pupae diet.

Regarding the β diversity (unweighted UniFrac distance), significant differences were observed between treatment and control groups (PERMANOVA <0.05, Figure 2). Nonetheless, weighted UniFrac distance showed no significant differences among hen groups (Figure 3).

**Figure 4:**
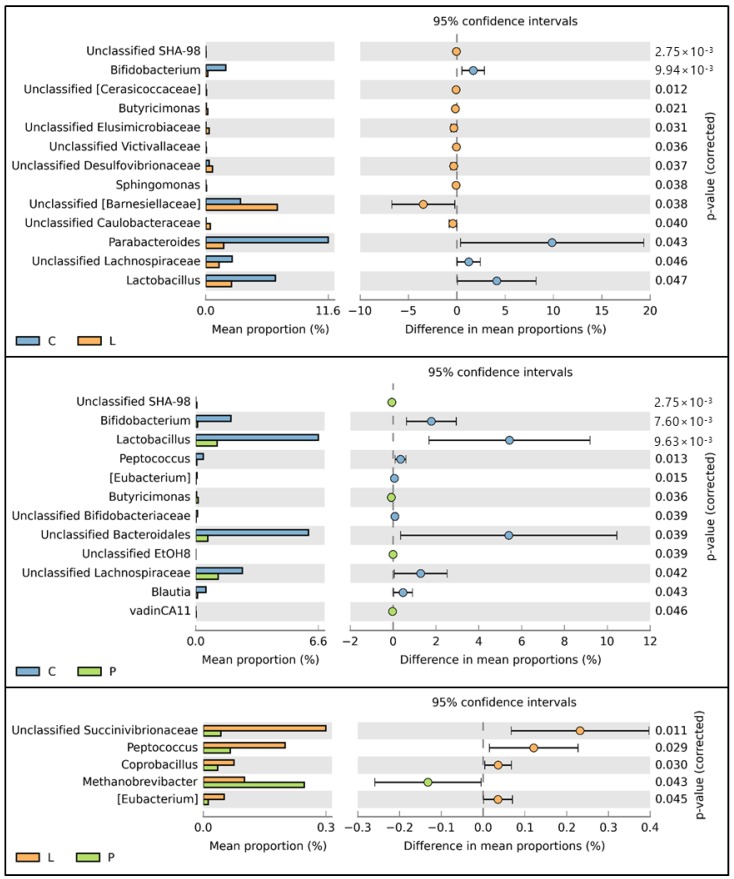
Comparative analysis of the taxonomic composition of microbial communities at the genus level (C: *n* = 9; L: *n* = 10; P: *n* = 10). Representative genera with significant differences between groups are indicated by the histograms and P value determinations, as calculated by STAMP software. C: control diet; L: larvae diet; P: pre-pupae diet.

The abundance of 13, 12, and five genera significantly differed between C and L groups, C and P groups, and L and P groups, respectively (Figure 4).

## 4. Discussion

In the present work, soybean meal (47.4%) and soybean oil (100%) of the control diet were replaced by BSF larvae or pre-pupae in the experimental diets. BSF oil did not affect body weight and the egg laying rate of laying hens, or the height of villi and depth of crypts in their small intestine. In contrast, previous studies [8,9] reported a reduction of feed intake and body weight in poultry due to the inclusion of BSF into the diets. Thus, although BSF larvae and pre-pupae oil could potentially replace soybean oil in poultry diets [20], in practice, caution should be taken to ensure that all fatty acids, with emphasis on linoleic acid, are provided at adequate levels.

It is well known that villi and crypts in the small intestine contribute to the digestion and absorption of dietary nutrients [28]. Moreover, previous studies have reported that feeding indigestible carbohydrates such as mannanoligosaccharides to poultry increases villus height [29]. Therefore, in the present study, we considered that feeding BSF-derived chitin would also increase villus height. However, unexpectedly, no differences in the villus height or crypt depth were detected among hen groups.

An et al. [30] found that an increase of calcium levels in diets resulted in an improvement of eggshells’ strength in laying hens. In the present study, although no significant differences in eggshell strength were found among groups, eggshell thickness was significantly greater in the P group when compared with the other groups. Nys [31] observed that dietary factors such as vitamin D, magnesium and dietary electrolytes affected eggshell quality. Interestingly, although the experimental diets of the present study had similar calcium contents, the calcium concentration in plasma of the P group was found to be higher than that of the L group. Hence, although the reason for this discrepancy remains unclear, it can be speculated that a high concentration of cecal SCFA in BSF pre-pupae may have contributed to an increased absorption of dietary minerals, calcium included, which subsequently caused an increase of eggshell thickness. Previous studies reporting that SCFA production is strongly related with Ca absorption in the distal colon [32,33], and this fact seems to support our hypothesis.

Currently, no information is available on the effect of feeding whole BSF larvae and pre-pupae meal on the cecal microbiota of laying hens. In the present work, Chao1 index was significantly higher in the P group than in the C group. Therefore, although we believe that BSF pre-pupae acted as fermentable matter in the cecum of hens, the concentration of SCFA in the cecal content remained unchanged among hen groups. However, since the total concentration of SCFA in the cecal content of the P group was higher than in the other groups, it can be suggested that some indigestible material derived from BSF pre-pupae underwent fermentation. In β diversity, although no significant differences in the weighted UniFrac distance were found among hen groups, unweighted UniFrac distances was indeed significantly different among groups. Hence, it can be suggested that feeding whole BSF larvae and pre-pupae affected certain microbial populations in the cecum, in particular in the cecal microbiota of BSF pre-pupae-fed hens. Apart from the Shannon index and the weighted UniFrac distance, cecal microbiota diversity in the present study was comparable with that reported in a previous study [15]. 

Regarding the bacterial genera in the gut of hens, the *Bifidobacterium* and *Lactobacillus* population was significantly lower in the treatment groups than in the control group. Indeed, the abundance of bacterial genera between treatment and control groups was different from that previously reported [15]. Although it remains unclear the reason why lactobacilli and bifidobacteria decreased in the treatment groups, BSF fat can be suggested as the possible cause. In the present experiment, no morbidity or mortality was observed. In addition, *Campylobacter* and *Salmonella*, known to cause food poisoning, remained unchanged among treatment groups. Nonetheless, lactobacilli and bifidobacteria are considered probiotic bacteria that modulate the intestinal microbiota and inhibit pathogens [34,35,36,37], improve the immune response [38,39] and induce growth [40] in poultry. Thus, to compensate for the decreased lactobacilli and bifidobacteria in the gut of hens, the addition of beneficial bacteria to feed in which BSF larvae and pre-pupae have substituted other ingredients, may be necessary. Finally, the content of saturated fatty acids in diets seems to affect the intestinal microbiota greater than does the content of protein [41]. However, feeding a diet with high contents of lauric acid and myristic acid to chickens did not affect the microbial population in their gut **[42]**. Therefore, it is likely that the effect of saturated fatty acids on the intestinal microbiota in poultry depends on the type of saturated fatty acid added to diets.

## 5. Conclusions

In summary, it was demonstrated that BSF larvae and pre-pupae raised on household organic waste could potentially be used as ingredients for poultry diets (≤10% of total feed). However, further investigation is recommended to determine the highest possible level of BSF oil that can be added to poultry diets, paying close attention to the total fat content of the diet and its fatty acid composition. In addition, the effect of feeding BSF larvae and pre-pupae to poultry should be further investigated to detect any undesirable effect on the nutritional composition or flavor of meat and eggs. 

## Figures and Tables

**Table 1:** Ingredients in the experimental household waste.

Ingredients	Concentration (w/w %)
Cabbage	17
Potato	16
Carrot	17
Apple pomace	5
Grapefruit pomace	4
Orange pomace	4
Banana peel	5
Ground pork	8
Eggshell	2
Horse mackerel	10
Rice	3
Bread	3
Wheat noodle	3
Chinese noodle	3
**Total**	**100**

**Table 2:** Ingredients and chemical composition of the experimental diets.

Ingredients (%)	C	L	P
Maize grain	68.0	68.0	68.0
Soybean meal	19.0	10.0	10.0
Larvae	0.0	10.0	0.0
Pupa	0.0	0.0	10.0
Soybean oil	1.0	0.0	0.0
Tricalcium phosphate	1.0	1.0	1.0
Calcium carbonate	9.0	9.0	9.0
Salt	1.0	1.0	1.0
Vitamin and mineral mix	1.0	1.0	1.0
**Chemical composition (% as feed) and energy content (Kcal/kg)**			
Dry matter ^a^	89.6	90.5	90.6
Crude protein ^a^	14.4	14.6	16.5
Crude fiber ^a^	2.3	2.8	3.0
ADFom ^a^	4.3	5.1	4.8
NDFom ^a^	17.6	20.8	20.8
Ether extract ^a^	3.5	3.7	5.1
Ash ^a^	12.7	14.7	14.8
Ca ^a^	3.6	3.5	3.3
P ^a^	0.5	0.5	0.5
Mg ^a^	0.2	0.2	0.2
Chitin ^b^	-	0.39	0.67
Gross energy ^b^ (kcal/kg)	3159.4	3103.0	3169.3

C: Control diet; L: larvae diet; P: pre-pupae diet. ^a^ Analyzed data; ^b^ Calculated data.

**Table 3:** Amino-acid concentration (% of dry matter) of black soldier fly larvae and pre-pupae.

Amino Acid	Larvae	Pre-Pupae
Arginine	1.94	2.20
Alanine	2.45	2.28
Aspartic acid	3.43	3.74
Cysteine	0.28	0.28
Glutamic acid	3.99	4.30
Glycine	1.90	2.11
Histidine	1.32	1.30
Isoleucine	1.57	1.71
Leucine	2.59	2.81
Lysine	2.22	2.51
Methionine	0.58	0.74
Phenylalanine	1.51	1.69
Proline	2.16	2.14
Serine	1.62	1.70
Threonine	1.42	1.55
Tryptophan	0.53	0.66
Tyrosine	2.30	2.63
Valine	2.25	2.38
Total	34.06	36.73

**Table 4:** Fatty acid concentration (% of dry matter) of black soldier fly larvae and pre-pupae.

Fatty Acid	Larvae	Pre-Pupae
C10:0	0.7	0.8
C12:0	14.1	16.1
C14:0	1.9	1.8
C16:0	5.3	4.2
C16:1	1.1	1.0
C18:0	0.9	0.6
C18:1n-7	0.2	0.1
C18:1n-9	7.3	5.6
C18:2n-6	2.7	2.5
C18:3n-3	0.2	0.2
C20:0	0.0	0.0
C20:1	0.0	0.0
C20:5n-3	0.3	0.3
C22:0	0.0	0.0
Saturated fatty acids (SFA)	22.9	23.5
Monounsaturated fatty acids (MUFA)	8.6	6.7
Polyunsaturated fatty acids (PUFA)	3.2	3.0

**Table 5:** Feed intake and production parameters of laying hens (*n* = 18).

Item	C	L	P
Feed intake (g/day)	79.8 ± 0.6	77.5 ± 1.3	76.2 ± 4.9
Egg-laying rate (%)	70.3 ± 5.5	70.6 ± 6.3	70.7 ± 3.1
Egg weight ^c^ (g)	48.0 ± 0.3 ^a^	49.0 ± 0.7 ^a^	51.1 ± 0.3 ^b^
Feed requirement rate	2.5 ± 0.1	2.3 ± 0.2	2.2 ± 0.1
Body weight ^c^ (g/bird)	1230.7 ± 166.6	1234.6 ± 124.4	1328.7 ± 98.1
Liver weight ^c^ (% of BW)	1.8 ± 0.1	1.8 ± 0.1	1.8 ± 0.1

C: control diet; L: larvae diet; P: pre-pupae diet. Data: mean ± SE, ^a,b^ *p* < 0.05. ^c^ Measured on day 35 of the experiment.

**Table 6:** Villus height, crypt depth and goblet cell density in the small intestine of laying hens in samples collected during the last day of the experiment (*n* = 10).

Item	C	L	P
Villus height			
Duodenum	1028.9 ± 51.8	993.2 ± 63.0	893.8 ± 48.4
Jejunum	604.6 ± 30.7	590.1 ± 23.4	561.2 ± 31.6
Ileum	318.4 ± 17.5	351.1 ± 26.0	321.7 ± 17.2
Crypt depth			
Duodenum	103.9 ± 3.2	107.0 ± 8.8	98.1 ± 3.1
Jejunum	63.3 ± 4.1	62.1 ± 2.6	63.5 ± 2.4
Ileum	59.9 ± 3.0	66.1 ± 2.3	58.4 ± 4.4
Villus height/Crypt depth			
Duodenum	10.1 ± 0.2	10.2 ± 0.8	9.5 ± 0.5
Jejunum	9.9 ± 0.5	9.9 ± 0.4	9.2 ± 0.5
Ileum	5.5 ± 0.2	5.5 ± 0.3	6.1 ± 0.4

Data: mean ± SE.

**Table 7:** Egg quality of laying hens at day 30 of the experiment (*n* = 18).

Item	C	L	P
Egg weight (g)	45.66 ± 1.15 ^a^	45.61 ± 0.88 ^a^	49.91 ± 0.60 ^b^
Egg yolk weight (g)	12.02 ± 0.31	12.03 ± 0.25	12.56 ± 0.24
Albumin weight (g)	26.79 ± 0.81 ^a^	27.10 ± 0.63 ^a^	30.04 ± 0.46 ^b^
Egg shell weight (g)	6.86 ± 0.30	6.49 ± 0.29	7.32 ± 0.23
Egg shell thickness (mm)	0.38 ± 0.02 ^ab^	0.37 ± 0.02 ^a^	0.43 ± 0.01 ^b^
Egg shell strength (kgf/cm^2^)	3.69 ± 0.33	3.64 ± 0.30	4.44 ± 0.17
Egg yolk color score	5.21 ± 0.30 ^a^	6.28 ± 0.22 ^b^	5.92 ± 0.36 ^ab^
Albumin height (mm)	7.16 ± 0.32 ^a^	7.64 ± 0.14 ^ab^	8.08 ± 0.18 ^b^
Haugh unit	88.59 ± 1.94	91.70 ± 0.69	92.66 ± 0.93

C: control diet; L: larvae diet; P: pre-pupae diet. Data: mean ± SE, ^a,b^ *p* < 0.05.

**Table 8:** Concentrations of calcium, inorganic phosphorus and magnesium (mg/dL) in plasma of laying hens (*n* = 18).

Mineral	C	L	P
Calcium	23.1 ± 1.7 ^ab^	17.6 ± 1.6 ^a^	24.6 ± 2.3 ^b^
Inorganic phosphorus	2.1 ± 0.1	1.7 ± 0.1	2.3 ± 0.1
Magnesium	2.7 ± 0.1	1.7 ± 0.1	2.7 ± 0.4

C: control diet; L: larvae diet; P: pre-pupae diet. Data: mean ± SE, ^a,b^ *p* < 0.05.

**Table 9:** Short chain fatty acids (SCFA) in the cecal content of laying hens at the last day of the experiment (*n* = 10).

SCFA (µg/L)	C	L	P
Acetic acid	87.4 ± 17.3	110.2 ± 13.0	95.4 ± 11.0
Propionic acid	41.7 ± 7.7	64.9 ± 9.7	53.1 ± 5.9
n-butyric acid	204.9 ± 83.5	197.5 ± 45.7	276.6 ± 138.7
Total	334.0 ± 102.5	372.5 ± 61.2	425.1 ± 142.1

C: control diet; L: larvae diet; P: pre-pupae diet. Data: mean ± SE.
